# Association between non-high-density lipoprotein cholesterol to high-density lipoprotein cholesterol ratio (NHHR) and gout in US adults: a cross-sectional study of the mediating role of BMI

**DOI:** 10.1186/s13098-025-01798-2

**Published:** 2025-06-18

**Authors:** Hongmei Li, Xu Li, Wei Zhong, Fangjiu Liu

**Affiliations:** 1 Department of Blood Transfusion, Suining Central Hospital, Suining, Sichuan China; 2 Department of Vascular Surgery, Suining Central Hospital, Suining, Sichuan China

**Keywords:** NHHR, NHANES, Gout, Body mass index

## Abstract

**Background:**

The connection between non-high-density lipoprotein to high-density lipoprotein ratio (NHHR) and gout has been documented among American adults; however, the specific mechanisms underlying this relationship remain to be elucidated. This cross-sectional study aimed to explore the mediating role of Body mass index (BMI) in the relationship between NHHR and gout prevalence among US adults, using the National Health and Nutrition Examination Survey (NHANES) data.

**Methods:**

Participants were drawn from the NHANES across four cycles for this cross-sectional analysis. NHHR is characterized by the proportion of cholesterol that is non-high-density lipoprotein cholesterol to high-density lipoprotein cholesterol. We employed bootstrapping-based mediation analysis to assess the impact of NHHR on gout risk mediated by BMI.

**Results:**

The prevalence of gout in our study was found to be 5.07%. Multivariate logistic regression analysis identified a significant correlation between NHHR and the likelihood of developing gout (OR = 1.13, 95%CI 1.06–1.20, *p* = 0.001). Mediation analysis indicated that the relationship between NHHR and gout risk was partially mediated by BMI, accounting for 26.27% (95% CI 10.96–57.95%; p < 0.0001; total effect = 0.0076, direct effect = 0.0056) of the association.

**Conclusion:**

BMI significantly mediates part of the relationship between NHHR and gout among American adults, underscoring the need to factor in body weight when comprehending gout risk elements.

**Supplementary Information:**

The online version contains supplementary material available at 10.1186/s13098-025-01798-2.

## Introduction

Gout is a metabolic disease caused by a disorder of purine metabolism [1]. Severe gout patients may experience recurrent severe painful arthritis, joint damage, and even induce chronic diseases such as cardiovascular disease, chronic kidney disease, venous thromboembolism, and diabetes mellitus [2, 3]. Estimates suggest that approximately 41 million individuals globally are afflicted by the disease, with its incidence showing a rise [4]. Gout combined with metabolic syndrome adds to the global burden. It is therefore imperative to investigate the risk factors associated with the onset of gout. Emerging studies have consistently linked elevated NHHR to gout risk. For instance, Guo et al. reported an 18% increase in gout odds per 1-unit rise in NHHR (OR = 1.18, 95% CI 1.10–1.27) using NHANES data [5]. Mechanistically, NHHR reflects atherogenic dyslipidemia, which exacerbates lipid-driven inflammation and oxidative stress—key contributors to uric acid crystallization and gout pathogenesis [6, 7]. The relationship between the NHHR and gout, is a complex interplay that warrants detailed exploration.

NHHR (non-high-density lipoprotein cholesterol to high-density lipoprotein cholesterol) serves as an indicator of the balance between atherogenic and anti-atherogenic lipoproteins, demonstrating superior capability for metabolic disorders when compared to traditional lipid indices [8, 9]. An elevated NHHR is significantly associated with type 2 diabetes and impaired uric acid excretion, both of which are established risk factors for the development of gout [10–12]. Importantly, emerging evidence indicates that BMI may mediate these associations through two interconnected pathways: (1) inflammation of visceral adipose tissue leading to the overproduction of interleukin-6 (IL-6) and tumor necrosis factor-alpha (TNF-α), thereby enhancing systemic urate synthesis [13]; (2) obesity-induced insulin resistance resulting in reduced renal clearance of urate [14]. Notably, Not only has BMI been shown to be associated with hyperuricemia, but the risk of gout also increases in people with a BMI ≥ 30 kg/m^2^ [12, 15]. Given that BMI significantly influences hyperuricemia and gout, we question whether its mediating effect applies to the relationship between nhhr and gout.

In conclusion, although direct evidence linking NHHR and gout remains scarce, existing literature regarding nhhr’s involvement in metabolic disorders, combined with the mediating role of BMI in related diseases, implies a potential association. Nevertheless, to date, no pertinent reports have been published. Although NHHR's link to metabolic disorders (e.g., diabetes) was established [10], its pathway to gout—particularly through BMI-mediated mechanisms—remains unexamined due to: (1) prior focus on single lipid metrics rather than ratios [16]; (2) the research on the mediating role of BMI in the pathophysiology of gout in NHHR is limited. Consequently, a cross-sectional analysis of the NHANES data from 2011 to 2018 was conducted to explore the extent to which BMI mediates the association between NHHR and gout among American adults.

## Methods

### Study population

**Fig. 1:**
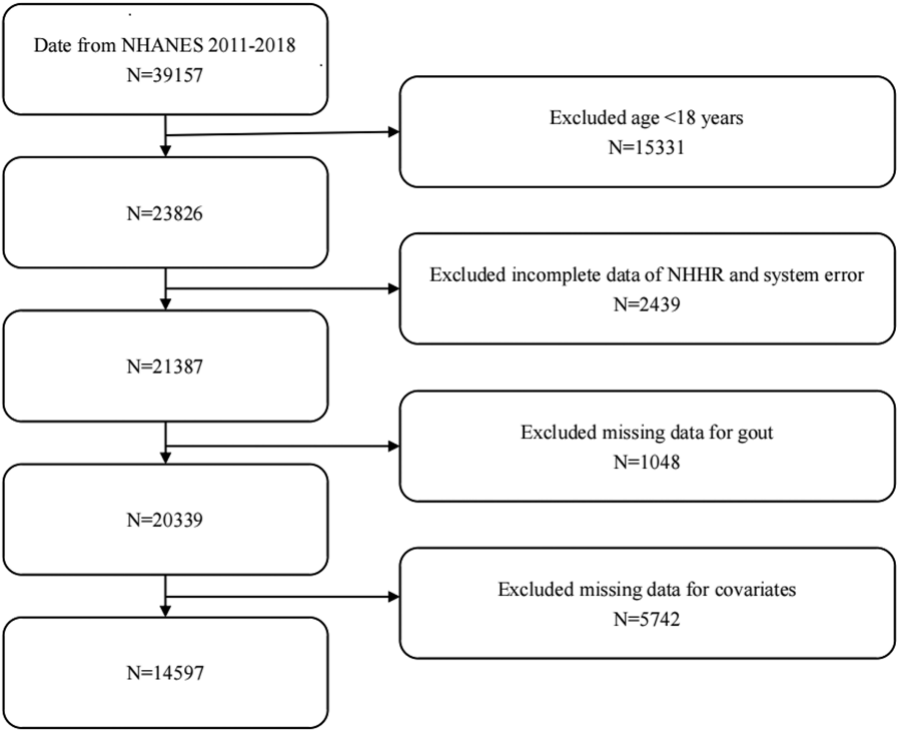
Flowchart of participant selection. Flowchart of participant selection; NHANES, National Health and Nutrition Examination Survey; NHHR, non-high-density lipoprotein cholesterol to high-density lipoprotein cholesterol ratio

NHANES, a comprehensive database that assesses the nutritional status and health-related conditions in the U.S. All participants were enrolled in the study following approval by the Ethics Committee of the National Center for Health Statistics and the provision of informed consent. All studies utilizing data can be accessed through the NHANES website at https://www.cdc.gov/nchs/nhanes/. Spanning from 2011 to 2018, the NHANES consists of four distinct phases, encompassing a sum of 39,157 individuals. Criteria for exclusion are outlined below: (1) age < 18 years, (2) lack of NHHR data (total cholesterol or high-density lipoprotein data) and system error, (3) those with missing data on gout, (4) covariates missing. The final participant count in this cross-sectional study was 14,597 (Fig. 1).

### The calculation of NHHR

NHHR is equal to non-high-density lipoprotein cholesterol (NHDL-C) divided by high-density lipoprotein cholesterol (HDL-C) [17], Which involves HDL-C directly from laboratory data in NHANES, and the value of NHDL-C corresponds to the variance between serum total cholesterol and HDL-C.

### Definition of gout

In this study, the presence of a history of gout was considered as an indicator of outcome, which was available in the NHANES questionnaire (MCQ160n), for the question Doctor ever told you that you had gout? answering yes was considered that they person had a history of gout, otherwise not.

### Covariates

Based on published articles, we selected these covariates. The demographic variables encompass age, sex, race, educational attainment, marital status and household income poverty rate (PIR). The questionnaire comprises questions pertaining to smoking status, the use of alcohol, hypertension (yes/no), diabetes (yes/no/borderline), weak/failing kidneys (yes/no) and physical activity. The inquiry “Have you smoked at least 100 cigarettes in your lifetime?” is indicative of the act of smoking,“Alcohol use” was judged by “Ever have 4/5 or more drinks every day?”. The health-related variables included BMI. The eGFR was computed via the 2009 CKD-EPI equation using laboratory data [18], and the energy intake is calculated by taking the average of two values from two 24-h recalled interviews. In addition, the usage of uric acid-lowering drugs, diuretics and lipid-lowering drugs was also recorded from the questionnaire data.

### Statistical analysis

Due to the complex stratified sampling methodology of the NHANES survey, sample weights were included for analysis in accordance with the NHANES guidelines in order to provide a more realistic representation of the overall situation. The research utilized survey-weighted linear regression for continuous variable analysis and employed survey-weighted Chi-square tests for categorical variable evaluation. A weighted logistic regression model was employed to examine the correlation between NHHR and gout, utilising NHHR as a continuous and categorical variable, respectively. In order to control for confounding factors, We comprehensively screened confounding factors based on the following three methods: (1) evaluation based on clinical significance, (2) assessment informed by existing literature [3, 19, 20], and (3) statistical analysis examining variations in the effect size of NHHR. Finally, Three different models were constructed for analysis. Model 1 was not adjusted, Model 2 was adjusted for age, gender, and race, and Model 3 was adjusted for PIR, education level, marital status, smoking, alcohol consumption, hypertension, diabetes, kidney disease, energy intake, physical activity, estimated glomerular filtration rate (eGFR), urate-lowering drugs, lipid-lowering drugs and diuretics on the basis of Model 2. And the association between NHHR and gout was further explored by fitting a smoothed curve through a generalized additive model. Finally, a stratified analysis was conducted to explore potential differences among specific populations, including variables such as age, gender, race, PIR, education level, marital status, smoking status, alcohol consumption, hypertension, diabetes, kidney disease, energy intake, physical activity, eGFR, and the use of urate-lowering drugs, lipid-lowering drugs, diuretics and uric acid.

Finally, Mediation analysis was employed to assess the mediating effects of BMI on the associations between the NHHR and gout, adjusting for the aforementioned variables. The presence of a mediating effect was determined by meeting all of the following criteria: a significant indirect effect, a significant total effect, and a positive proportion of the mediator effect [21, 22]. Multiple imputation techniques were employed to address the issue of missing data. All statistics in this study were evaluated using EmpowerStats (version 4.2) and R software (version 4.2.0), and bi-directional tests with p-values less than 0.05 were deemed to hold statistical significance.

## Results

### Characteristics of participants

Table 1 presents the findings of an analysis of data from 14,597 enrolled subjects, divided into two groups based on their history of gout, encompassing four NHANES cycles from 2011 to 2018.There were 740 (5.07%) with gout and 13,857 (94.93%) without gout. The NHHR in patients with gout was markedly elevated in comparison to that observed in patients without gout [3.20 (3.00–3.39) vs 2.83 (2.79–2.87), *p* = 0.004]. Notable disparities were noted between the pair of groups with regard to age, race, alcohol consumption, hypertension, diabetes, kidney disease, physical activity, eGFR, urate-lowering drugs, lipid-lowering drugs, diuretics, HDL-C, TC and NHHR (*p* < 0.05).

**Table 1:** Weighted baseline characteristics of participants

Characteristics	Non-Gout	Gout	*p*-value
Number	13,857	740	
Age (years)	47.17 (46.52,47.82)	60.52 (59.32,61.71)	< 0.001
Gender			< 0.001
Male	49.61 (48.68,50.55)	70.02 (64.59,74.94)	
Female	50.39 (49.45,51.32)	29.98 (25.06,35.41)	
Race			< 0.001
Mexican American	8.06 (6.49,9.97)	3.51 (2.32,5.27)	
Other Hispanic	5.61 (4.57,6.86)	3.62 (2.35,5.52)	
Non-Hispanic White	69.12 (65.74,72.30)	74.78 (69.56,79.36)	
Non-Hispanic Black	9.82 (8.20,11.70)	11.03 (8.68,13.91)	
Other race	7.40 (6.55,8.34)	7.07 (5.14,9.66)	
BMI (kg/m^2^)			< 0.001
< 18.5	1.58 (1.32,1.88)	0.61 (0.26,1.43)	
≥ 18.5, < 25	27.86 (26.47,29.30)	12.14 (9.40,15.54)	
≥ 25, < 30	32.23 (31.07,33.42)	30.95 (25.77,36.66)	
≥ 30	38.33 (36.83,39.85)	56.30 (49.96,62.44)	
PIR			0.392
< 1.3	20.21 (18.43,22.12)	20.77 (16.61,25.64)	
≥ 1.3, < 3.5	35.43 (33.65,37.26)	32.20 (28.07,36.63)	
≥ 3.5	44.35 (41.55,47.19)	47.04 (41.76,52.38)	
Marital status			< 0.001
Never married	18.59 (17.04,20.26)	7.22 (5.03,10.24)	
Married/Livingwithpartner	63.31 (61.43,65.16)	69.91 (65.34,74.12)	
Widowed/Divorced/Separated	18.09 (17.00,19.23)	22.87 (18.62,27.76)	
Education level			0.9360
Under high school	11.89 (10.60,13.31)	12.21 (9.87,15.00)	
High school grad or equivalent	22.29 (20.79,23.87)	22.71 (18.71,27.27)	
Some college or above	65.82 (63.49,68.08)	65.09 (59.66,70.16)	
Drinking status			< 0.0001
Yes	15.27 (14.17,16.44)	27.02 (23.30,31.10)	
No	84.73 (83.56,85.83)	72.98 (68.90,76.70)	
Smoking status			< 0.0001
Yes	47.01 (45.35,48.68)	61.36 (55.76,66.67)	
No	52.99 (51.32,54.65)	38.64 (33.33,44.24)	
Hypertension			< 0.0001
Yes	31.62 (30.21,33.07)	67.57 (62.35,72.39)	
No	68.38 (66.93,69.79)	32.43 (27.61,37.65)	
Diabetes			< 0.0001
Yes	9.52 (8.91,10.17)	25.71 (21.62,30.27)	
No	88.15 (87.40,88.86)	70.39 (65.12,75.16)	
Borderline	2.33 (1.96,2.76)	3.90 (2.33,6.46)	
Weak/failing kidneys			< 0.0001
Yes	2.32 (2.04,2.64)	11.10 (7.58,15.97)	
No	97.68 (97.36,97.96)	88.90 (84.03,92.42)	
Energy intake(kcal/day)			0.3094
	2141.38 (2119.66,2163.10)	2088.75 (1987.80,2189.70)	
Physical activity(MET/week)			< 0.0001
< 600	12.68 (11.92,13.48)	16.26 (13.37,19.65)	
≥ 600	67.07 (65.83,68.28)	55.54 (50.77,60.22)	
Missing	20.26 (19.09,21.48)	28.19 (23.90,32.92)	
eGFR (ml/min/1.73 m^2^)			< 0.0001
≤ 60	6.47 (5.92,7.07)	20.50 (16.64,24.98)	
> 60	93.53 (92.93,94.08)	79.50 (75.02,83.36)	
Urate-lowering drugs			< 0.0001
Yes	0.14 (0.08,0.25)	29.26 (24.48,34.54)	
No	99.86 (99.75,99.92)	70.74 (65.46,75.52)	
Lipid-lowering therapy			< 0.0001
Yes	16.80 (15.69,17.97)	37.28 (32.03,42.84)	
No	83.20 (82.03,84.31)	62.72 (57.16,67.97)	
Diuretics			< 0.0001
Yes	11.32 (10.63,12.05)	29.57 (25.34,34.19)	
No	88.68 (87.95,89.37)	70.43 (65.81,74.66)	
HDL-C (mmol/L)	1.40 (1.39,1.42)	1.25 (1.19,1.32)	< 0.0001
TC (mmol/L)	4.98 (4.94,5.01)	4.84 (4.72,4.97)	0.0468
NHHR	2.83 (2.79,2.87)	3.20 (3.00,3.39)	0.0004

Baseline characteristics of US adults stratified by presence of gout, NHANES 2011–2018. Continuous variables are expressed as weighted means (95% CI), and categorical variables are expressed as weighted percentages (95%CI); BMI: body mass index; PIR: family income-poverty ratio; eGFR: estimated glomerular filtration rate. HDL-C: high-density lipoprotein cholesterol; TC: total cholesterol; NHHR: non-high-density lipoprotein cholesterol to high-density lipoprotein cholesterol ratio

### The association between NHHR and gout

Table 2 demonstrates the relationship between NHHR and the incidence of gout, utilising a weighted multiple logistic regression model test as the statistical methodology. The analysis of the three models with different adjustment variables showed that NHHR was positively correlated with the possibility of gout occurrence. Specifically, Model 3, which was adjusted for multiple variables, suggests that there is an 13% increased risk of developing gout for every unit increase in NHHR (OR = 1.13, 95% CI 1.06–1.20, *p* = 0.0001). Furthermore, when NHHR was analysed as a categorical variable (based on quartile), it was found that there was a 0.42-fold increased risk of developing gout among individuals in the fourth vs. the first quartile (OR = 1.42,95% CI 1.10–1.85, *p* = 0.0077). A trend analysis was conducted using the median NHHR of each group as a continuous variable, which demonstrated that elevated NHHR levels were associated with an increased risk of gout. (*p* for trend = 0.0045).

**Table 2:** The association between NHHR and gout

Exposure	Model 1	Model 2	Model 3
	OR (95%CI) *P*-value	OR (95%CI) *P*-value	OR (95%CI) *P*-value
NHHR	1.12 (1.06, 1.17) < 0.0001	1.16 (1.10, 1.23) < 0.0001	1.13 (1.06, 1.20) 0.0001
Stratified by NHHR quartiles			
Quartile 1	Reference	Reference	Reference
Quartile 2	0.99(0.79, 1.23) 0.9052	0.98 (0.78, 1.23) 0.8537	0.95 (0.73, 1.25) 0.7252
Quartile 3	1.16 (0.93, 1.43) 0.1894	1.19 (0.95, 1.49) 0.1222	1.04 (0.80, 1.36) 0.7550
Quartile 4	1.46 (1.19, 1.79) 0.0003	1.60 (1.29, 2.00) < 0.0001	1.42 (1.10, 1.85) 0.0077
* P* for trend	< 0.0001	< 0.0001	0.0045

Model 1: unadjusted, Model 2: adjusted for age, sex and race, Model 3: based on Model 2, Model 3 further adjusted for PIR, education level, marital status, smoking, alcohol consumption, hypertension, diabetes, kidney damage, energy intake, physical activity, eGFR, urate-lowering drugs, lipid-lowering drugs and diuretics;

PIR: family income-poverty ratio; eGFR: estimated glomerular filtration rate; HDL-C: high-density lipoprotein cholesterol; TC: total cholesterol; NHHR: non-high-density lipoprotein cholesterol to high-density lipoprotein cholesterol ratio; OR: odds ratio; CI: confidence intervals

**Fig. 2:**
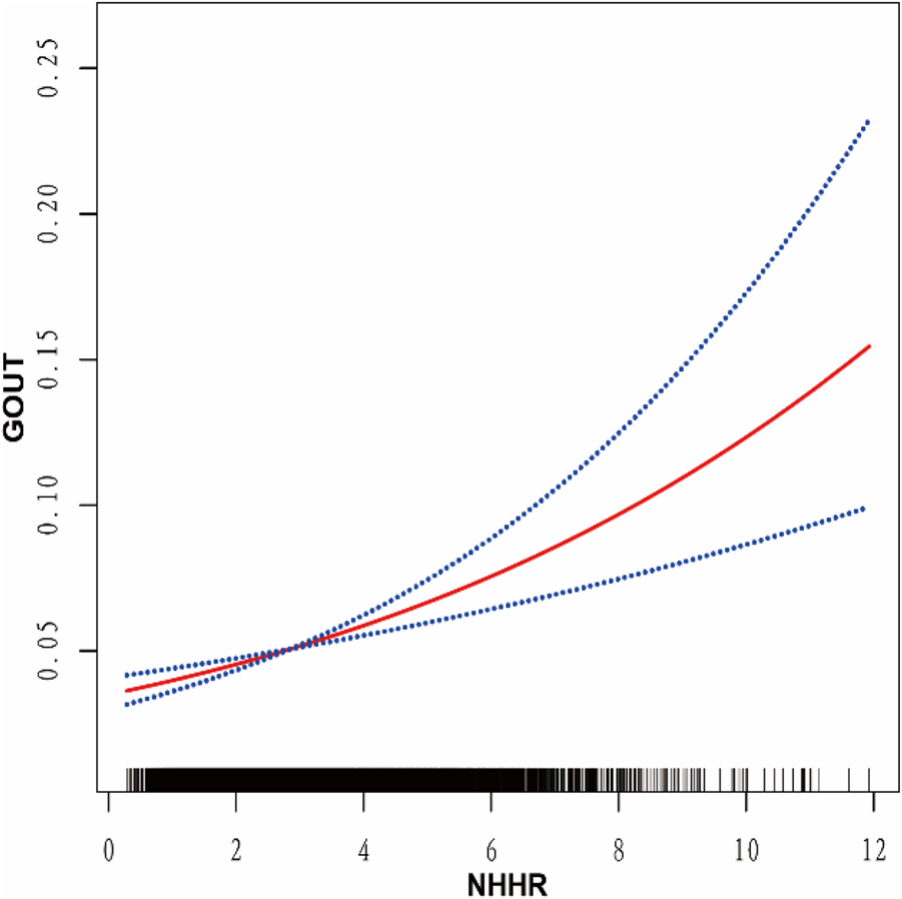
The relationship between NHHR and Gout. The relationship between NHHR and Gout is illustrated by the smoothed curve fitting method of the generalized additive model. The solid red line indicates the smoothed fitted curve for the variable and the 95% confidence interval is indicated between the blue dashed lines; NHHR, non-high-density lipoprotein cholesterol to high-density lipoprotein cholesterol ratio

Furthermore, In accordance with model 3, we employed smoothed curve fitting to explore how NHHR is related to the incidence of gout. As illustrated in Fig. 2, the smooth curve demonstrated a linear positive relationship between NHHR and gout risk.

### Multiple logistic regression of the associations of BMI with NHHR and gout

As demonstrated in Table 3, an examination of the relationship between NHHR and BMI reveals a positive correlation after adjusting for all variables (β = 1.32, 95%CI 1.24–1.40, *p* < 0.0001). Table 4 shows a significant association between BMI and gout prevalence, with a consistent increase in gout risk across all three models (*p* < 0.001).

**Table 3:** The associations between NHHR with BMI

Exposure	Model 1	Model 2	Model 3
	β (95%CI) *P*-value	β (95%CI) *P*-value	β (95%CI) *P*-value
NHHR	1.20 (1.11, 1.28) < 0.0001	1.40 (1.31, 1.48) < 0.0001	1.32 (1.24, 1.40) < 0.0001
NHHR categorical			
Q1	Reference	Reference	Reference
Q2	2.39 (2.07, 2.71) < 0.0001	2.64 (2.33, 2.95) < 0.0001	2.57 (2.27, 2.86) < 0.0001
Q3	3.93 (3.61, 4.25) < 0.0001	4.36 (4.05, 4.67) < 0.0001	4.16 (3.86, 4.46) < 0.0001
Q4	4.80 (4.48, 5.12) < 0.0001	5.59 (5.27, 5.91) < 0.0001	5.35 (5.04, 5.66) < 0.0001
* P* for trend	< 0.0001	< 0.0001	< 0.0001

Model 1: unadjusted, Model 2: adjusted for age, sex and race, Model 3: based on Model 2, Model 3 further adjusted for PIR, education level, marital status, smoking, alcohol consumption, hypertension, diabetes, kidney damage, energy intake, physical activity, eGFR, urate-lowering drugs, lipid-lowering drugs and diuretics;

BMI: body mass index; NHHR: non-high-density lipoprotein cholesterol to high-density lipoprotein cholesterol ratio; CI: confidence intervals

**Table 4:** The associations between BMI with gout

Exposure	Model 1	Model 2	Model 3
	OR (95%CI) *P*-value	OR (95%CI) *P*-value	OR (95%CI) *P*-value
BMI	1.04 (1.03, 1.05) < 0.0001	1.06(1.05,1.07) < 0.0001	1.03(1.02, 1.04) < 0.0001
BMI categorical(kg/m^2^)			
< 18.5	Reference	Reference	Reference
≥ 18.5, < 25	0.73 (0.36, 1.45) 0.3672	0.67 (0.33, 1.37) 0.2699	0.60 (0.28, 1.29) 0.1918
≥ 25, < 30	1.24 (0.63, 2.44) 0.5414	0.99 (0.49, 1.99) 0.9687	0.75 (0.36, 1.59) 0.4585
≥ 30	1.71 (0.87, 3.36) 0.1195	1.74 (0.87, 3.50) 0.1185	1.01 (0.48, 2.14) 0.9741
* P* for trend	< 0.0001	< 0.0001	0.0002

Model 1: unadjusted, Model 2: adjusted for age, sex and race, Model 3: based on Model 2, Model 3 further adjusted for PIR, education level, marital status, smoking, alcohol consumption, hypertension, diabetes, kidney damage, energy intake, physical activity, eGFR, urate-lowering drugs, lipid-lowering drugs and diuretics

BMI: body mass index; OR: odds ratio; CI: confidence intervals

### Mediating role of BMI

**Fig. 3:**
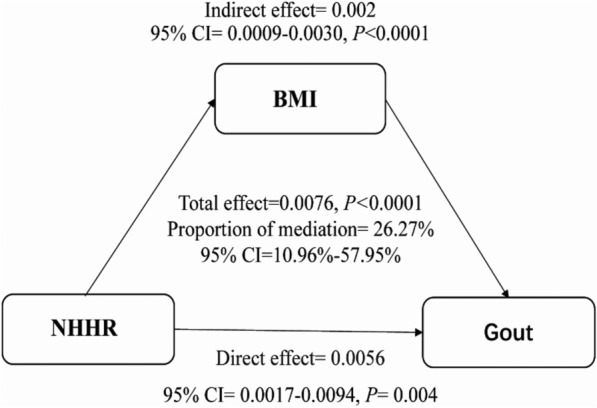
The mediating effect of BMI on the relationship between NHHR and gout. Adjusted for age, sex, race, PIR, education level, marital status, smoking, alcohol consumption, hypertension, diabetes, kidney damage, energy intake, physical activity, eGFR, urate-lowering drugs, lipid-lowering drugs and diuretics; BMI: body mass index; NHHR: non-high-density lipoprotein cholesterol to high-density lipoprotein cholesterol ratio; CI: confidence intervals

As is shown in Fig. 3, After controlling for all possible confounders, The analysis demonstrated that elevated NHHR significantly increased gout risk through both direct and BMI-mediated pathways. The total effect of NHHR on gout was 0.0076 (p < 0.0001), with average mediation effect was 0.0019 (95% CI 0.0009–0.0030; p < 0.0001), Among them, approximately 26.27% (95%CI 10.96–57.95%, p < 0.0001) attributed to BMI-mediated mechanisms, while the remaining 73.73% (average direct effect = 0.0056, 95% CI 0.0017–0.0094; p = 0.004) reflected NHHR’s direct biological influence independent of BMI. Figure 3 illustrates this dual pathway, indicating that the impact of nhhr on the risk of gout not only stems from its direct influence but is also related to BMI-related metabolism.

### Subgroup analyses and sensitivity analyses

A subgroup analysis was performed to assess the stability of the association between NHHR and gout. As illustrated in Supplementary Fig. 1, the results remained consistent when stratified by variables such as age, gender, race, BMI, PIR, educational level, marital status, smoking status, alcohol consumption, hypertension, diabetes, kidney damage, energy intake, physical activity, eGFR, use of uric acid-lowering drugs, lipid-lowering drugs, diuretics, and uric acid levels. In terms of sensitivity analysis, two approaches were undertaken. First, due to the left-skewed distribution of NHHR, a natural logarithmic transformation was applied. The mediation analysis results concerning BMI and the effect of NHHR on gout were consistent with the original data, as presented in Supplementary Table 1. Second, the mediation analysis results after multiple imputation of missing data, shown in Supplementary Table 2, also indicated consistency. These analyses collectively demonstrate the robustness of our findings.

## Discussion

This study is the first to identify that BMI may partially mediate the relationship between NHHR and gout in American adults (n = 14,597). The findings reveal a significant positive correlation between NHHR and the incidence of gout, potentially operating through dual pathways, 73.73% attributable to the direct biological effect and 26.27% to an indirect BMI-mediated mechanism.

The ratio of NHDL-C to HDL-C (NHHR) serves as a comprehensive biomarker that reflects the dynamic equilibrium between atherogenic and atheroprotective lipoprotein particles. HDL-C exerts cardioprotective effects by facilitating the removal of excess cholesterol from peripheral tissues through reverse cholesterol transport, thereby mitigating the risk of atherosclerosis [23, 24]. Conversely, NHDL-C encompasses apolipoprotein B-containing lipoproteins that contribute to cholesterol deposition, endothelial damage, and inflammation [25, 26]. An elevated NHHR indicates a pro-atherogenic state characterized by an imbalance between deleterious NHDL-C and beneficial HDL-C. This imbalance is mechanistically associated with metabolic syndrome, insulin resistance, and hyperuricemia, which are critical pathways in the pathogenesis of gout [5, 27, 28]. The reason why the mediating role of BMI in the association between NHHR and gout has not been studied may be a methodological challenge. Prior research has indicated a positive correlation between NHHR and the risk of diabetes, and with a significant association observed in the increased risk of depression. As we all know, diabetes has been established as an independent risk factor for gout; Furthermore, depressive states may indirectly elevate the risk of gout by influencing a patient's lifestyle and metabolic status [9, 29, 30]. This is consistent with our research result that NHHR is positively correlated with gout.

Elevated NHHR has been demonstrated to contribute to an increased risk of gout through two distinct pathways: firstly, a direct pathway involving disturbances in lipid metabolism associated with hyperuricemia; and secondly, an indirect pathway mediated by BMI. For the direct pathway, the dysregulation of lipid profiles associated with increased NHHR—particularly elevated levels of triglycerides and LDL—may further facilitate urate crystal deposition by increasing vascular permeability and oxidative stressit [31]. Is imperative to acknowledge the significance of two additional factors that exert a substantial influence on the propensity to develop gout. These factors encompass the concept of fatty toxicity and genetic elements associated with NHHR. It is possible that all of the aforementioned elements may collectively constitute the part that is not BMI-mediated [32].

Our research shows that approximately 26.27% (95% CI 10.96–57.95%; p < 0.0001) of the impact of NHHR on the risk of gout is mediated by BMI. This partial mediation parallels findings in other metabolic diseases: for example, obesity mediates 30–40% of the TyG index's effect on hyperuricemia in hypertensive adults, and visceral adiposity amplifies insulin resistance in erectile dysfunction via inflammatory cytokines [33, 34]. Notably, this tissue-specific duality of BMI is further underscored by evidence suggesting that while BMI exacerbates gout risk through inflammatory pathways, indices like TyG-BMI incorporating BMI may concurrently exert protective effects on bone mineral density, likely due to BMI-driven mechanical loading [35]. In gout, this phenomenon may be associated with the inflammatory response induced by BMI, particularly in the context of visceral adipose tissue. This kind of inflammation leads to the excessive production of pro-inflammatory cytokines. Elevated BMI may contribute to a systemic inflammatory environment through lipid-mediated activation of the NLRP3 inflammasome [36–38]. induces cytokine-driven inflammation, including interleukin-6 and tumor necrosis factor-alpha exacerbating insulin resistance and impairing renal urate excretion (e.g., downregulation of ABCG2/URAT1), ultimately leading to hyperuricemia and gout thereby intensifying inflammation induced by monosodium urate crystals [39–41]. This inflammatory response associated with high BMI results in the excessive production of pro-inflammatory cytokines also can augment systemic urate synthesis, a pivotal element in the pathogenesis of gout [42–44]. In addition, the systemic elevation of these cytokines can lead to increased hepatic production of urate, thereby heightening the risk of hyperuricemia [12].

Furthermore, the decreased clearance rate of renal uric acid represents a significant factor in understanding how obesity influences the risk of gout. A higher BMI has consistently been linked to an elevated risk of hyperuricemia [45]. Obesity is often accompanied by insulin resistance, which can impair renal capacity to effectively excrete uric acid [46, 47]. This impairment in renal function results in increased serum uric acid levels, thereby creating a conducive environment for gout attacks. For example, research has demonstrated that the accumulation of visceral fat, due to increased production of free fatty acids and other metabolites affecting renal function, is associated with a reduced urate clearance rate by the kidneys [48, 49]. To summarise, there is a multifaceted interplay between NHHR, BMI, and the onset of gout. BMI functions as a pivotal intermediary in this relationship.

Our research presents several advantages. Firstly, we utilized the nationally representative NHANES database, which offers a large and reliable sample size. The application of sample weights in our analysis ensures a more accurate representation of the U.S. population. Secondly, we rigorously addressed potential confounding variables, thereby enhancing the reliability of our results. Third, the mediating role of BMI in gout was proposed for the first time. Nonetheless, our research is not without its limitations. Firstly, limiting the sample to American adults may restrict the applicability of our results to different groups, necessitating further research to assess broader applicability. Secondly, while we controlled for some confounding factors, not all variables influencing the association between NHHR and gout may have been accounted for, including unmeasured chronic diseases, reverse causation remains possible (e.g. gout-related inflammation may alter lipid profiles or weight). Future longitudinal studies are needed to confirm temporal relationships. Thirdly, despite the relatively low Variance Inflation Factor values for NHHR and BMI, it is important to acknowledge the potential influence of residual confounding from shared metabolic pathways, such as insulin resistance, on the mediation analysis. Fourthly, the reliance on self-reported data from questionnaires in the NHANES database as the diagnostic criterion for gout may introduce biases. After considering the missing values, the core results are consistent. Fifthly, the cross-sectional nature of the data used in this study excludes inferences that causal pathways and relationships cannot be derived. Future studies should explore the integration of BMI with uric acid metabolism parameters and inflammatory biomarkers as potential mediating variables, and investigate the association between NHHR and the incidence of gout.

## Conclusion

This study reveals a positive relationship between NHHR and gout incidence, linked by BMI, among the US population over 18 years. It is proposed that incorporating lipid profiles and BMI into the clinical evaluation framework may represent a novel approach for assessing the risk of gout.

## Supplementary Information

Supplementary Material 1.

## Data Availability

The datasets used and/or analysed during the current study are available from the corresponding author on reasonable request.

## Abbreviations

NHANES: National Health and Nutrition Examination Survey
NHHR: Non-high-density lipoprotein cholesterol to high-density lipoprotein cholesterol ratio
NHDL-C: Non-high-density lipoprotein cholesterol
HDL-C: High-density lipoprotein cholesterol
BMI: Body mass index
LDL: Low-density lipoprotein
VLDL: Very-low-density lipoprotein
TC: Total cholesterol
TG: Triglyceride
PIR: Family income–poverty ratio
eGFR: Estimated glomerular filtration rate
IL-6: Interleukin-6
TNF-α: Tumor necrosis factor-alpha
OR: Odds Ratio
CI: Confidence Intervals
