# Supplementary material for: Association between non-high-density lipoprotein cholesterol to high-density lipoprotein cholesterol ratio (NHHR) and gout in US adults: a cross-sectional study of the mediating role of BMI
Source: Diabetol Metab Syndr. 2025 Jun 18;17:223. doi: 10.1186/s13098-025-01798-2 (PMC12175315; doi:10.1186/s13098-025-01798-2)
Supplement: Supplementary file 1 — Supplementary Material 1. [file 13098_2025_1798_MOESM1_ESM.docx]

**Supplementary material**

**Supplementary Figure**

**Supplementary Figure 1. Forest plot for subgroup analysis of association between NHHR and gout.**


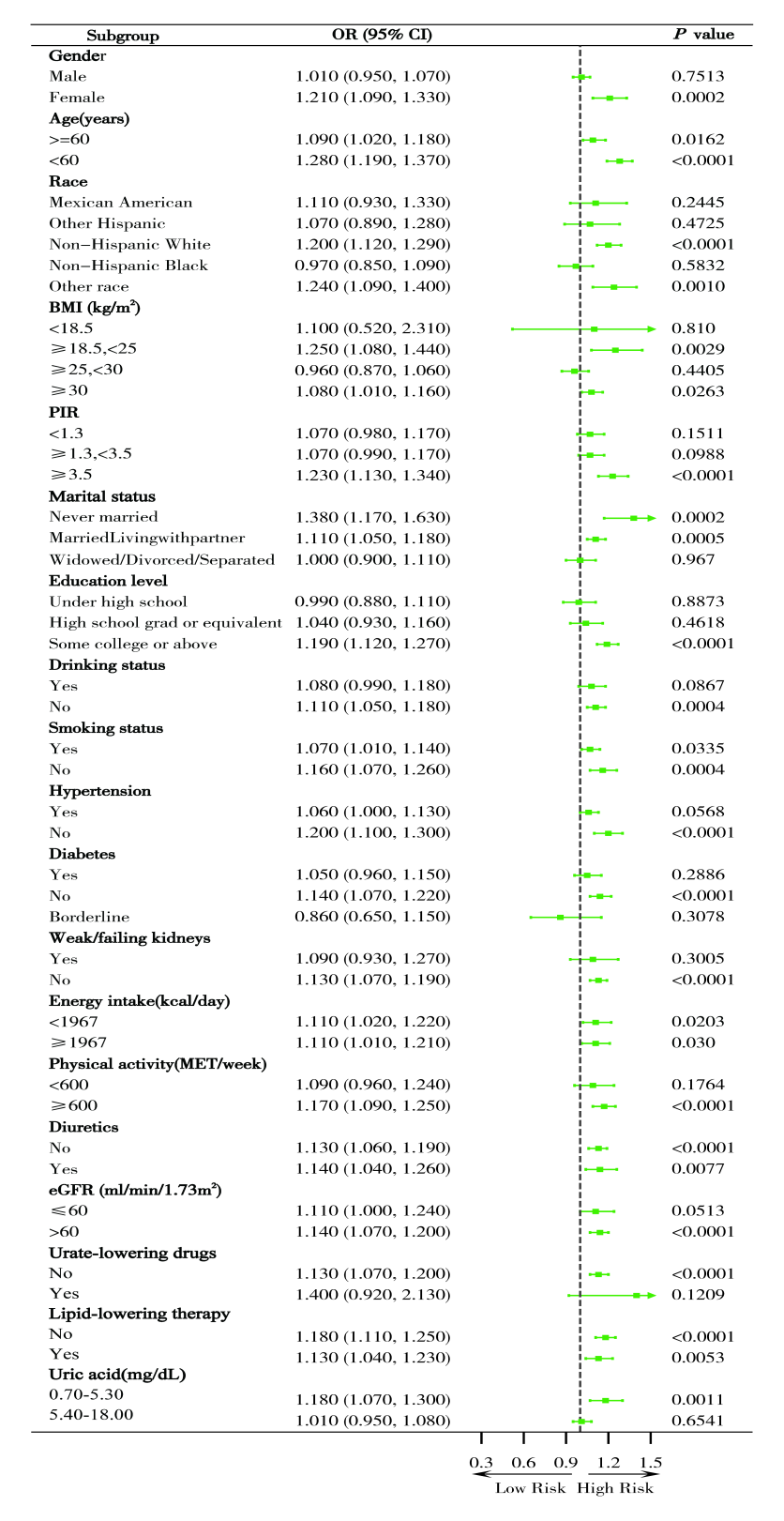


BMI, body mass index; PIR, education level, eGFR,estimated glomerular filtration rate; OR, odds ratio; CI, confidence intervals.

**2. Supplementary Table**

**Supplementary Table 1. The mediating effect of BMI on the relationship between NHHR and gout after natural logarithmic transformation of NHHR**

|  | Estimate | 95% CI lower | 95% CI upper | P-value |
| --- | --- | --- | --- | --- |
| Total effect | 0.007795 | 0.003315 | 0.012276 | <0.0001 |
| Mediation effect (average) | 0.002566 | 0.001232 | 0.003853 | <0.0001 |
| Direct effect (average) | 0.005229 | 0.000710 | 0.010227 | 0.0240 |
| Propotion mediated (average) | 0.329204 | 0.139420 | 0.796130 | <0.0001 |

Adjusted for age, sex, race, PIR, education level, marital status, smoking, alcohol consumption, hypertension, diabetes, kidney damage, energy intake, physical activity, eGFR, urate-lowering drugs, lipid-lowering drugs and diuretics; BMI, body mass index; NHHR, non-high-density lipoprotein cholesterol to high-density lipoprotein cholesterol ratio; CI, confidence intervals.

**Supplementary Table 2. The mediating effect of BMI on the relationship between NHHR and gout after** **multiple imputations(5 times) of missing data.**

**multiple imputation 1**

|  | Estimate | 95% CI lower | 95% CI upper | P-value |
| --- | --- | --- | --- | --- |
| Total effect | 0.007482 | 0.003736 | 0.010893 | <0.0001 |
| Mediation effect (average) | 0.001956 | 0.000895 | 0.002976 | <0.0001 |
| Direct effect (average) | 0.005526 | 0.001654 | 0.009326 | 0.0040 |
| Propotion mediated (average) | 0.261430 | 0.109405 | 0.586441 | <0.0001 |

**multiple imputation 2**

|  | Estimate | 95% CI lower | 95% CI upper | P-value |
| --- | --- | --- | --- | --- |
| Total effect | 0.007482 | 0.003744 | 0.010901 | <0.0001 |
| Mediation effect (average) | 0.001955 | 0.000893 | 0.002979 | <0.0001 |
| Direct effect (average) | 0.005527 | 0.001659 | 0.009328 | 0.0040 |
| Propotion mediated (average) | 0.261345 | 0.109362 | 0.586357 | <0.0001 |

**multiple imputation 3**

|  | Estimate | 95% CI lower | 95% CI upper | P-value |
| --- | --- | --- | --- | --- |
| Total effect | 0.007481 | 0.003735 | 0.010894 | <0.0001 |
| Mediation effect (average) | 0.001957 | 0.000894 | 0.002978 | <0.0001 |
| Direct effect (average) | 0.005524 | 0.001655 | 0.009320 | 0.0040 |
| Propotion mediated (average) | 0.261570 | 0.109470 | 0.588807 | <0.0001 |

**multiple imputation 4**

|  | Estimate | 95% CI lower | 95% CI upper | P-value |
| --- | --- | --- | --- | --- |
| Total effect | 0.007489 | 0.003741 | 0.010905 | <0.0001 |
| Mediation effect (average) | 0.001954 | 0.000893 | 0.002979 | <0.0001 |
| Direct effect (average) | 0.005536 | 0.001666 | 0.009334 | 0.0040 |
| Propotion mediated (average) | 0.260839 | 0.109101 | 0.585112 | <0.0001 |

**multiple imputation 5**

|  | Estimate | 95% CI lower | 95% CI upper | P-value |
| --- | --- | --- | --- | --- |
| Total effect | 0.007484 | 0.003733 | 0.010904 | <0.0001 |
| Mediation effect (average) | 0.001955 | 0.000893 | 0.002978 | <0.0001 |
| Direct effect (average) | 0.005529 | 0.001658 | 0.009320 | 0.0040 |
| Propotion mediated (average) | 0.261254 | 0.109570 | 0.586952 | <0.0001 |

Adjusted for age, sex, race, PIR, education level, marital status, smoking, alcohol consumption, hypertension, diabetes, kidney damage, energy intake, physical activity, eGFR, urate-lowering drugs, lipid-lowering drugs and diuretics; BMI, body mass index; NHHR, non-high-density lipoprotein cholesterol to high-density lipoprotein cholesterol ratio; CI, confidence intervals.
